# Parents’ perspectives of the transition to home when a child has complex technological health care needs

**DOI:** 10.5334/ijic.1852

**Published:** 2015-09-29

**Authors:** Maria Brenner, Philip J. Larkin, Carol Hilliard, Des Cawley, Frances Howlin, Michael Connolly

**Affiliations:** School of Nursing, Midwifery & Health Systems, University College Dublin, Dublin, Ireland; Clinical Nursing (Palliative Care), Our Lady's Hospice and Care Services, University College Dublin, Dublin, Ireland; Nurse Practice Development Coordinator, Our Lady's Children's Hospital, Crumlin, Dublin, Ireland; Department of Nursing and Health Science, Athlone Institute of Technology, Athlone, Ireland; School of Nursing, Midwifery & Health Systems, University College Dublin, Dublin, Ireland; School of Nursing, Midwifery and Health Systems, University College Dublin, Dublin, Ireland

**Keywords:** complex care, children, transitional care, homecare

## Abstract

**Introduction:**

There is an increasing number of children with complex care needs, however, there is limited evidence of the experience of families during the process of transitioning to becoming their child's primary care giver. The aim of this study was to explore parents’ perspectives of the transition to home of a child with complex respiratory health care needs.

**Methods:**

Parents of children with a tracheostomy with or without other methods of respiratory assistance, who had transitioned to home from a large children's hospital in the last 5 years, were invited to participate in the interviews. Voice-centred relational method of qualitative analysis was used to analyse parent responses.

**Results:**

Four key themes emerged from the interviews including “stepping stones: negotiating the move to home”, “fighting and frustration”, “questioning competence” and “coping into the future”.

**Discussion:**

There is a need for clear and equitable assessments and shared policies and protocols for the discharge of children with complex care needs. Direction and support are required at the level of health service policy and planning to redress these problems. This study provides evidence that the transition of children with complex care needs from hospital to home is a challenging dynamic in need of further improvement and greater negotiation between the parent and health service provider. There are tangible issues that could be addressed including the introduction of a standardised approach to assessment of the needs of the child and family in preparation for discharge and for clear timelines and criteria for reassessment of needs once at home.

## Background

Recent population prevalence figures show that there is an increasing number of children with complex care needs [1, 2]. While there are many definitions of complex care, it is generally accepted that children with a medical complexity have substantial health care needs as a result of one or more chronic conditions, with functional limitations that often require technology assistance and need to access multiple health support services [3]. Much research in this area has identified specific physical and emotional needs of parents caring for a child with complex health needs in the home [4–6], while a recent integrative literature review identified critical factors for a successful transition to home [7]. These included effective multi-agency agreements, robust clinical governance, effective discharge planning, effective care packages, individually tailored family support, accessible accommodation and well-functioning acute community care relationships. This review suggests that, in general, advances in medical health care that enable children with complex care needs to survive for longer are often not mirrored with advances in supportive health systems which accommodate the transition of such children to being cared for at home. It is acknowledged that the term transition is also used in the context of children transferring to adult care services; however, it is increasingly being used internationally to reflect the discharge to home of children with complex health care needs from acute care services; most commonly from transitional care units within these facilities.

There are some robust examples of systems of care delivery that may be facilitative of the health care demands of children and families transitioning to the home environment, such as the multi-professional primary care teams in Sweden and the Netherlands [8]. Other countries, such as Italy, have a strong focus on the continuum of care across the acute and community services [9]. Anecdotal evidence from an Irish perspective suggests that the health service may be struggling to meet the demands of these families. On discharge to home families of children are generally provided with a “homecare package”, an arrangement for care support for the child and family. However, this is often on ad hoc basis as there is no nationally agreed framework for the assessment of the needs of these families, no agreed pathway for integrated care for this population and the process for seeking funding for these packages is often reliant on parents’ persistence and lobbying. There is limited evidence of the actual experience of families during the process of transitioning to becoming their child's primary care giver. Little is known about their readiness to assume this role or their experience of the peri-discharge period. The aim of this study was therefore to explore Irish parents’ perspectives of the transition of a child with complex respiratory health care needs, from hospital to home, which could help inform the national model of care for the discharge to home of this group.

## Methods

### Study design

A qualitative approach employing narrative analysis was used in this study to elicit the experiential stories of parents with the intent of moving from the explanation from what actually happened to making sense of what happened. We conducted interviews with parents to portray the emic perspective of this experience, the “insider” perspective of transitioning to home by those directly involved and impacted by this experience, thereby facilitating a deconstruction and exploration of their experiences.

### Study sample

Parents of children with a tracheostomy with or without other methods of respiratory assistance, who had transitioned to home from a large children's hospital in the last 5 years, were invited to participate in the study. Following research ethical approval from the participating hospital and the associated university, potential participants were identified by the clinical nurse managers. Parents of a child who were currently having a medical crisis were not invited to participate.

Potential participants were provided with detailed written information which included information on the aim, purpose and significance of the study and the appropriate time commitment required. Prospective participants were advised that participation was voluntary and they were encouraged to contact one of the researchers (M.B.) if they had any queries or would like to take part in the study.

A telephone interview was subsequently conducted with parents over a six month period in 2013. Parents were asked three open-ended questions which included asking them to recall what enabled their transition to home, any difficulties they encountered in making the transition to caring for their child at home and any concerns they had about caring for their child with complex care needs in the home. While we found that parents were very eager to speak of their experiences we did use prompts, where needed, to encourage parents to elaborate more on a point of significant interest. Typical prompts included Can you elaborate on what you mean? Can you tell me more about that? Can you give me more detail on that specific issue? Each interview was recorded using a confidential teleconferencing system and lasted between 60 and 90 minutes. Interviews concluded with the researcher summarising the main points as well as offering the participants the opportunity to add any additional comments.

### Data analysis

Interview recordings were transcribed verbatim. Voice-centred relational method of qualitative analysis was used to analyse parent responses. This involved examining the data for the plot, the voice of the “I”, relationships and the cultural contexts and social structures of the participants [10], subsequently identifying themes and sub-themes. In choosing this method of analysis we were recognising that data analysis was not a discrete phase, rather it began when we first accessed the sample, continued as we listened to the stories we were told during the interviews and made decisions about which issues to follow up and probe further. Therefore, all researchers who were involved in data collection were also involved in the data analysis.

The initial reading of the transcripts involved looking for when parents used the first person pronoun; this was important to get a sense of how they personally reflected on their journey of transitioning to home with their child and gave a sense of how they perceived their own identity during this process. In reading for relationships we looked at how the parents spoke about others, those they frequently mentioned and those they never mentioned, while differentiating between those they spoke well of and those that they perceived had a negative impact on their transition to home experience. Finally, when reading for cultural contexts and social structures we paid attention to how the parents felt they were treated as a parent of a child with complex care needs. Our research group gathered after each reading where we also read extracts from each others’ interviews. Although this was time-consuming, it was extremely beneficial in affording us the opportunity to point out anything that may have been missed or anything that another member regarded as a key point. Throughout this process we were seeking the themes and sub-themes that represented the central ideas of meaning for participants as a whole.

Rigour for the study was established using core principles applied within qualitative research [11]. Credibility of the data was established by the inclusion of parents who had experience of transitioning to home with a child with complex care needs; this also addressed the criterion of authenticity, as an understanding of the constructs of others was sought through evidence of a variety of realities contained in the data collected. Accuracy of the data collected was established through the verbatim transcripts of the audio files, while confirmability of the data was supported by keeping an audit trail of conclusions reached. Transferability of data from the interviews was established by presenting detailed information on sampling, data gathering, analysis and interpretation when reporting the findings.

## Results

### Participants

A total of 15 parents participated in the interviews, 12 mothers and 3 fathers, representing the transition to home of 15 children. The majority of children were discharged to home with a homecare package. The evidence suggests that there was great variation in the length of time the children spent in hospital prior to discharge (3–22 months) (Table 1) as well as a difference in hours of care provided through individual homecare packages. Four key themes which emerged from the interviews were “stepping stones: negotiating the move to home”, “fighting and frustration”, “questioning competence” and “coping into the future”.

### Stepping stones: negotiating the move to home

Parents interviewed were very eager to discuss their experience of transitioning to home and in general were very positive about the beginning of the process of moving to caring for their child at home. Parents stated that nurses and other health care professionals delivered the best care to their child in the hospital setting and that they prepared them well for when they themselves became the primary caregiver:

…they helped us do our best to care for him within the hospital, which is really what the transitional care unit is about. It is about empowering parents and to assist them moving into caring for their kids. (P3)

One stepping stone to home in this unit included phased responsibility for care, whereby there was a gradual transition towards parents taking responsibility for their child's care, including parents taking their child home for a few hours or overnight. Parents found this phased process very supportive in helping them get a sense of the reality of the level of organisation and management of care delivery that would be required for their child on eventual discharge, and how their plans for the full move to home would eventually be operationalised:

Before we came home fully they gave you stepping stones, we brought him out for a day and came back then and I think that happened twice. Leave the hospital, bring him out of the hospital and come back then for the night and maybe the next day we went out again. And eventually we left. (P11)

Parents highly valued this and found it gave them an opportunity to identify knowledge or skill gaps and then focus on developing their capability to adapt better on the next visit home with their child.

While all of the parents interviewed spoke of the value of the staged process to going home, they collectively identified themselves as overwhelmed when they were eventually discharged from hospital. Parents stated that they were often “petrified” or “terrified” in their initial days at home despite their perceived clinical readiness and sense of empowerment while in the hospital. Many expressed a sense of discomfort and uncertainty as they became the primary care giver on a permanent basis:

So that was a big step to being at home with her by yourself, being solely responsible for everything. (P4)

[In hospital] you have everyone around you, but when you are at home and it is the middle of the night and you are only awake and you awaken to a blocked tube, that is when it really hits home … because at the end of the day if it goes wrong, it goes wrong big time. (P6)

### Fighting and frustration

Each of the parents interviewed expressed their anger and frustration at the length of time it took to have everything in place so they could take their child home and how hard they had to fight to secure and retain homecare for their child:

That was the most frustrating thing and knowing that [son] was ready to get home. We could have been home weeks ago but trying to fight basically for hours for nursing at home. (P14)

Parents repeatedly found a lack of joined up thinking across all the service providers, including a perceived lack of equity in access to homecare packages, and sometimes delays of months between applying for and receiving this homecare. Each parent described the lengths they had to go to fight to get their child out of hospital. They emphasised the need to seek advocates beyond health care. This included repeated calls to the health service and lobbying local politicians:

So that was the frustrating part, waiting on funding for the package, that was really frustrating I have to say. You have got the costs of going in and out of the hospital and he was well enough to come home but you couldn't come home because you needed your care package. That was the most frustrating part I have to say. Yes he got it [tracheostomy] in April and I would say, April, May, June, July, by the end of August or the start of September we applied for the homecare package and it took until the end of November and that was with a lot of fighting. That was getting TDs [members of parliament in Ireland] and everything involved in it. (P10)

The piece that I found most frustrating on a consistent basis was that each parent was having to fight the same battles from scratch which to me is not just bad for the parents but a complete waste of time and resources on the healthcare side.(P8)

Parents stated that they often felt very stressed and isolated as they fought to get the health service to work with them to get their child home. This frustration was often compounded by repeated and intense questioning about their child's condition, with no formal assessment criteria applied:

…it was as if the person interviewing us was starting from complete scratch and had no context of our child or the condition or anything like that which was massively frustrating … it felt like a grilling for no real purpose or benefit. And that was just very, very frustrating. (P8)

Parents described how this frustration was often further compounded by responses they received to what they perceived to be reasonable arguments for assistance:

My wife was fighting our case saying it is costing more money to keep [son] in hospital and her answer was, “well a Dublin hospital is saving the health board here money.” That was their look at it, the longer [son] stays in hospital the less money they have to spend here locally. (P11)

While many of the parents interviewed stated that their protracted fighting eventually got them what they needed, others stated that they eventually accepted a deal for less hours than they needed “just to get home”. When this happened, parents stated that they were often left feeling exhausted from lack of sleep. They often referred to not feeling able to challenge the hours they got because it came after a very long battle with the health service and many gave practical examples, as depicted here:

I suppose my biggest issue with the nursing was the length of hours that we got, we either got a 7 hour or an 8 hour night. Obviously it is like with any hospital, the changeover, the nurses were normally doing 12:00 until 7:00 for us with community care. When they'd come at 12:00 we would have to spend about half an hour speaking how [son] was getting on through the day, what he needed, what he didn't need, any medical things that were going on through that day. So it would be nearly 1:00 before we would go to bed and then in order to have a shower in the morning I would have to get up at 6:00 to be ready for 7:00.So I was literally getting 5 hours sleep for a 7 hour night care so that was a major issue. (P4)

Once funding was in place for a homecare package and parents could take their child home they then described “the next phase of fighting”. This referred to the parents struggle to retain funding and their fears of resource reviews, particularly when they felt they had already been allocated limited resources:

they were reviewing funding for paediatrics in the community and they wanted to cut ours because at this stage she was probably coming up to 4 and was deemed to be not requiring the same amount of funding as a baby, that she should be a bit more independent … even the hospital didn't agree that anything had significantly changed in her situation that would deem it appropriate to cut the funding. (P4)

### Questioning competence

Many of the parents interviewed raised concern about the quality of care available in the community and in regional hospitals once they had been discharged from the tertiary care centre. With regard to community care, some parents raised concern about the way some health professionals reacted to their child, while the majority of parents perceived that many of these staff were unable to care for their child:

Nurses were afraid of her basically. They were afraid of her equipment, we had to be there all the time. You couldn't leave the room because they were afraid of her and that is my biggest, biggest problem. Even to this day when we take [daughter] in [to that hospital] there is a scatter the minute her name is mentioned, people are going, “oh God, here comes [daughter].” (P9)

This extended to concern about variations in the level of knowledge and skill of those who were sent to care for their child when the child was at home:

…I would come down during the middle of the night and find them asleep or something like that when they were supposed to be looking after her … it would be devastating and you'd be upset, but also very angry, the fact that this person would do that and take a risk with your child when you wouldn't even take that risk to fall asleep … which put us very on edge with anyone else that ever came in after that. You would always have to get up and check a couple of times during the night. (P4)

…the nurses need to understand what a child with a tracheostomy needs … we have had nurses come to our house and we just sent them away. They'd take one look at our child and run out the door, they just don't seem to get it. And to be honest I don't know how you would be able to get into people's heads how important it is to have someone who is competent, who comes in the door and tells you they can use all the equipment that needs to be used for your child and ends up having to call you downstairs in the middle of the night because they can't connect an oxygen tube into the back of a ventilator. Or can't adjust the suction power on the suction machine, although they tell you they can do all of this before they start their shift. (P9)

### Coping into the future

Parents spoke at length about their ongoing challenges and their hopes for the future. This included difficulty thinking beyond the present moment, concern for their child coping with their level of ability and the need for a compassionate health service to help parents cope. Many parents stated that they were very much coping on a day-to-day basis, fearful of losing hard-won services and doing their best to cope with being a full-time carer. There was often a long pause when asking about future concerns with parents, suggesting that the future could be very negative to contemplate:

I genuinely don't know about the future, it is not something that we let ourselves think about because it would make us more depressed. It is a very, very difficult situation to be in. (P1)

Others contemplated how their child might cope with living with complex care needs and how this might be a burden for the child:

…if he goes into his teenage years, into secondary school, with the tracheostomy and into work, all those years, will he ever get a job, will he go to college, will he be able to do these things or is he still going to have the tracheostomy and be held back? (P6)

The majority of parents interviewed expressed concern about lack of compassion from the health service and expressed hope that this might addressed in the future.

Compassion is the biggest thing, a little bit of compassion, a little bit of understanding. I do honestly think that everybody should be retrained again in the line of the compassion side. It is all well and good for a public health person to come to my door and say, “you should be doing this or you can have this but we don't have money for it.” They are saying it with no compassion, they don't care, they still get their wage at the end of the week and they go home (P5)

## Discussion

Four key themes emerged from our interviews with parents: “stepping stones: negotiating the move to home”, “fighting and frustration”, “questioning competence” and “into the future”.

A particular strength of this study is that it is representative of parents from a wide geographical spread across Ireland. This means that these parents offered a unique perspective on the process of transitioning to home to a variety of health service areas.

Overall, parents interviewed in this study were very positive about the care they received in the acute care setting. They also stated that their expectation of being prepared in this environment for the subsequent clinical care of their child at home was very well met. This finding is not consistent with findings from previous studies which found that a common stressor for parents was a deficit in the quality and consistency of their training to care for their child and the subsequent negative impact of this on their ability to care for their child at home [12, 13]. There is also a higher readmission rate reported in the literature for children whose parents did not perceive that they understood how to manage their child's health after discharge to home [14]. This collectively supports the current training programme delivered to parents interviewed in this study.

Parents interviewed repeatedly expressed their frustration at the length of time it took to get home which suggests a lack of equity in the current approach to the assessment of needs of these families and suggests a lack of equity in the funding arrangements, to the point that parents had to lobby local representatives to progress their transition to home. Parents’ frustration at the process of transitioning is supported internationally, with finance agreements, recruiting, training and retaining appropriate community-based carers reported as frequently problematic [15–17]. In addition to the introduction of a standardised assessment tool and timely arrangement of a homecare package this issue could also be addressed by the introduction of a key worker; the introduction of this role has been found to enhance parents trust in the health service and reduce their time commitment to coordinating the care of their child in other jurisdictions [18, 19]. While this role is operationalised by some private health care agencies in Ireland it has not yet been evaluated; it is necessary to do this to understand the full contribution of this role in the Irish health care setting and to then consider the value of the full roll-out of this innovative practice.

Many of the parents interviewed raised concern about the quality of care delivery available in the community and in regional hospitals once they had been discharged from the tertiary care hospital. They felt burdened by the additional responsibility of supervising the care delivered by nurses or health care assistants in regional hospitals and in their own homes and making decisions about care delivery to their children. There is very limited mention of accountability concerns in the research on the transition of children with complex care needs from hospital to home. However it is known that traditionally health professionals have been dominant in decisions about planning and delivering care when a child is in hospital [20, 21]. There is evidence that parents want to be part of decision-making about care delivery and want to work with health care professions in the development and implementation of dynamic care plans for their child [22]. However there is no evidence to suggest that parents wish to take full responsibility for care decisions [23]. A challenge therefore occurs when parents are discharged to home and become the primary care giver of a child with complex care needs. The parents interviewed in this study identify a significant shift at this point to having greater responsibility for care delivery and the oversight of care delivered to their child with a perceived lack of support for the oversight of the quality of this care from any service body. The governance of care for children with complex care needs in the community in Ireland is often unclear and is compounded by the fragmented nature of health service delivery to this population. This approach requires direction and support at the level of health service policy and is compounded by the absence of nationally agreed standards for the care of children with health care needs in hospital or in the community in Ireland.

This problem is not unique to the Irish setting. It is consistent with the diverse approach to the education preparation of nurses and health care professionals who care for these children and their families across Europe and a distinct lack of consensus regarding the need to hold a qualification in children's nursing. This may be due to wide variation in the delivery of this education. In some countries children's nursing education programmes are offered as direct-access programmes, others offer it as a post-registration programme, while in eight countries in Europe nurses delivering care to children do not have any specific education to prepare them for practice [24].

One potential way forward would be to follow the model of care in the UK with the development of children's community nursing teams, established to enable children and young people to receive nursing care in their own homes, and to keep hospital admissions to a minimum by working with families to self-manage conditions. However, this has been described as a fractured system, with a lack of availability of such teams in some parts of the UK or insufficient service hours to adequately cover the care needs of some children [25]. To address this issue from an Irish perspective there is a need for direction at policy level, with agreement on the criteria for competent care delivery and clarity in the responsibility and regulation of training and education of nurses and health care staff caring for these children. This would facilitate understanding of the role of the carer and the competencies they possess to help the family care for the child's current needs or respond to any changing patterns of caring.

As the provision of care closer to home is increasingly recognised as an objective for care for children with complex care needs there is an increasing need for further research in to established practices to explore the level and extent of integration of services which are being provided. Such research must take into consideration any potential variance in service provision, for example between rural and urban areas, to identify any potential inequity arising due to geographic location. While appreciating that children encounter the world in an individual manner, the views of children with complex care needs or their siblings were not sought in this study as the specific aim for this study was to explore parents’ experience of transitioning to home. The majority of children with complex care needs were under 5 years of age with various communication difficulties, therefore it would not have been appropriate to invite their participation. It is imperative, where appropriate, to seek the views of children with complex care needs, and their siblings, about their experiences.

## Limitations

The use of telephone interviews as opposed to face-to-face interviews could be viewed as a limitation of this study. Some qualitative methodologists are not in favour of this approach suggesting that telephone interviews may be used as a time-saving exercise and that it may hinder the researcher's ability to build a rapport with the participants and thereby hinder the opportunity to gather in-depth data [26, 27]. This was not our experience. Our decision to interview parents by phone was based on our collective experience of collecting data with vulnerable populations and our acknowledgement of the fact that the parents we sought to speak with often struggled to get time for their own family life. Therefore we deliberately sought an approach that would not impose on them any more than was necessary. We communicated the rationale for our approach to the interviews with parents in our initial letter of invitation and all of the parents interviewed expressed their appreciation of our understanding of their time commitments. We found that this enhanced their trust in us as researchers and subsequently led to our ability to gather rich data.

## Conclusion

This study provides evidence that the transition of children with complex care needs from hospital to home is a challenging dynamic in need for further improvement and greater negotiation between the parent and health service provider. There are tangible issues that could be addressed including the need for system level measures to standardise the approach to assessment of the needs of the child and family in preparation for discharge and for clear timelines and criteria for reassessment of needs once at home. There is also a need to consider the need for specialist community based posts, including care coordinators and nurses with specialist training in this field to support the transition to home of these children and to support their ongoing care in the community.

## Figures and Tables

**Table 1.**
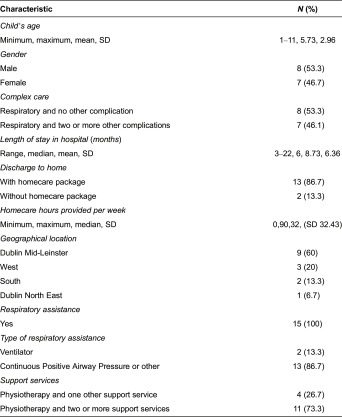
Profile of children discharged to home
